# SSDoH and Aging in Persons of African Ancestry

**DOI:** 10.1093/geroni/igab046.307

**Published:** 2021-12-17

**Authors:** Joyce Balls-Berry

**Affiliations:** Washington University School of Medicine Knight Alzheimer's Disease Research Center, Washington University in St. Louis, Missouri, United States

## Abstract

Persons of African Ancestry (Black) encompasses a broad spectrum of individuals across the African diaspora. The diversity of the Black community must be considered in the context of SSDoH especially as it relates to diseases of aging. Blacks report higher levels of discrimination as a barrier to Alzheimer’s Disease or related dementia (ADRD) care, are less likely to receive timely diagnoses of ADRD, and many do not trust that a future cure for ADRD will be shared equally and equitability with their community compared to their white counterparts. Once diagnosed, older Blacks, are twice as likely as their white counterparts to have ADRD. A key to addressing the Black community’s ADRD needs is speaking openly about the historical underpinnings related to social injustice and racism as a link to appropriate ADRD diagnoses. Ultimately, SSDoH impact treatment, healthcare policy, and the future of biomedical research for the Black community.

